# Multi-Layer Mechanical Model of Glagov Remodeling in Coronary Arteries: Differences between *In-Vivo* and *Ex-Vivo* Measurements

**DOI:** 10.1371/journal.pone.0159304

**Published:** 2016-07-18

**Authors:** Pak-Wing Fok

**Affiliations:** Department of Mathematical Sciences, University of Delaware, Newark, DE 19716, United States of America; Magna Graecia University, ITALY

## Abstract

When blood vessels undergo remodeling because of the buildup of atherosclerotic plaque, it is thought that they first undergo compensatory or outward remodeling, followed by inward remodeling: the lumen area stays roughly constant or increases slightly and then decreases rapidly. The second phase of remodeling is supposed to start after the plaque burden exceeds about 40%. These changes in the vessel were first observed by S. Glagov who examined cross-sections of coronary arteries at different stages of the disease. In this paper, we use a mathematical model based on growth and elasticity theory to verify the main aspects of Glagov’s result. However, both our model and curve-fitting to the data suggest that the critical stenosis is around 20% rather than 40%. Our model and data from the PROSPECT trial also show that Glagov remodeling is qualitatively different depending on whether measurements are taken *ex-vivo* or *in-vivo*. Our results suggest that the first outward phase of “Glagov remodeling” is largely absent for *in-vivo* measurements: that is, the lumen area always decreases as plaque builds up. We advocate that care must be taken when infering how *in-vivo* vessels remodel from *ex-vivo* data.

## Introduction

When arteries are compromised by atherosclerotic plaque, they undergo remodeling: persistent histological changes that are often accompanied by growth or resorption. The mechanisms that lead to various types of vessel remodeling have been studied by many researchers [1, 2], and the way that vessels remodel is vitally important to understanding the progression of atherosclerosis.

Atherosclerotic plaques can be symptomatic or asymptomatic [3]. Symptomatic plaques are often associated with encroached lumens which may precipitate shortness of breath, angina, and a visit to the doctor. However, many plaques are asymptomatic because blood vessels are able to maintain their flow capacity even in the presence of disease [4]. Patients with asymptomatic plaques are unlikely to seek out medical assistance if they do not present symptoms. Even if they do, such plaques are difficult to detect using angiography since this imaging modality provides mainly a visualization of the lumen. It is important to be able to diagnose asymptomatic plaques because they may pose equal or even greater risk than symptomatic ones [5].

An artery wall consists of three main layers. The innermost intima is usually very thin in healthy human arteries and consists of a layer of connective tissue. The next layer is called the media, containing many smooth muscle cells. The intima and media are separated by a thin layer of tissue called the internal elastic lamina (IEL). Finally the adventitia surrounds the media. Atherosclerosis is a complex disease that involves inflammation, deposition of lipoproteins and intima growth [6]. In many atherosclerotic arteries, the intima is considerably thicker than in healthy arteries, often presenting calcification and necrosis [7].

Medical researchers have tried to understand the morphological evolution of plaques by measuring two key geometric parameters. The first is the area of the IEL, the total area enclosed by the IEL. The second is the area of the lumen. Two related metrics of vessel remodeling are:

$$\begin{array}{ccc} \text{plaque}\;\text{area}\; & = & \text{IEL}\;\text{area}-\text{lumen}\;\text{area}, \end{array}$$ (1)

$$\begin{array}{ccc} \text{stenosis} & = & \frac{\text{Plaque}\;\text{area}}{\text{IEL}\;\text{area}}. \end{array}$$ (2)

Stenosis fraction quantifies how healthy an artery is. A fully occluded artery has a stenosis of 100% while an artery with no disease has a stenosis of 0%.

**Fig 1:**
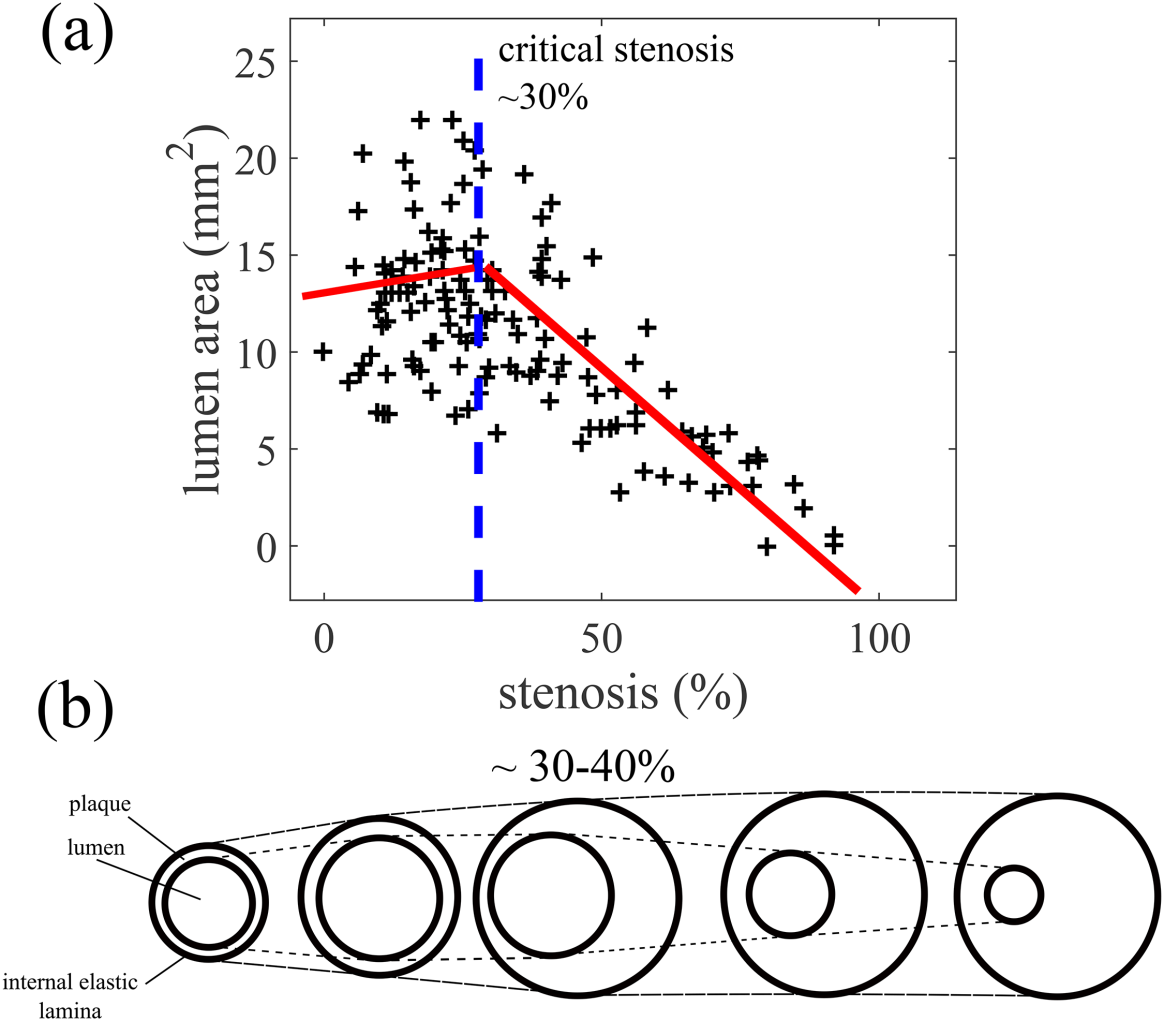
Glagov remodeling in atherosclerotic arteries. (a) Glagov et al. [4] analyzed the left main coronary artery in 136 hearts to deduce changes in morphology with respect to plaque burden. Remodeling behavior depends on whether stenosis is less than or greater than 30%. With the same data, the authors were also able to fit a piecewise linear curve with a jump in derivative at about 40% (not shown). In this case, the curve gently decreased when the stenosis was <40% and rapidly decreased when the stenosis was >40%. (b) In Glagov remodeling, initially, the lumen area increases slightly while the internal elastic lamina (IEL) increases in area. After the plaque reaches about 40% of the IEL area, the luminal area starts to decrease. Adapted from [4].

Over 25 years ago, Glagov et al. [4] published a seminal result on arterial modeling. By studying coronary arteries, the authors established one of the main quantitative results in atheroscelerosis: that vessels remodel in two stages, depending on the stenosis fraction. They arrived at this result by measuring the IEL areas, lumen areas, plaque areas and stenoses of 136 coronary arteries. They plotted the lumen area as a function of stenosis and in Fig 1(a), we qualitatively duplicate their best-fit curves. The researchers concluded that for small stenoses, the lumen area increases slowly with respect to stenosis fraction. For large stenoses, the lumen area rapidly decreases with respect to stenosis fraction. Fig 1(b) shows the sequence of events that is thought to occur when an atherosclerotic vessel remodels. In this paper, we call the stenosis value at which the switch in behavior occurs the *critical stenosis*. This value is thought to be at about 40%. Glagov reasoned that for stenoses less than the critical stenosis, the lumen roughly maintains its area as the plaque grows (compensation), but when the plaque burden exceeds 40%, one starts to see a rapid decrease in lumen area (inward remodeling). Glagov’s result has been reported for *in-vivo* imaging studies in humans [8] as well as *ex-vivo* in mice [9] However, to date there has not been a satisfactory explanation for Glagov’s observations. Why does remodeling occur in two stages? Why does the critical stenosis occur at 40% and not some other value?

In this paper, we attempt to put Glagov’s observations on a theoretical foundation by means of a mathematical model. The model assumes that the vessel is made up of three concentric hyperelastic annuli. The innermost annulus represents the intima and undergoes uniform growth while the vessel lumen is pressurized. The outer two annuli represent the media and adventitia and each of the three layers is characterized by different material properties. We find that this simple model can give rise to Glagov remodeling and that the qualitative nature of the remodeling is different depending on whether the lumen is pressurized (*in-vivo*) or not (*ex-vivo*).

## Mathematical Model

**Fig 2:**
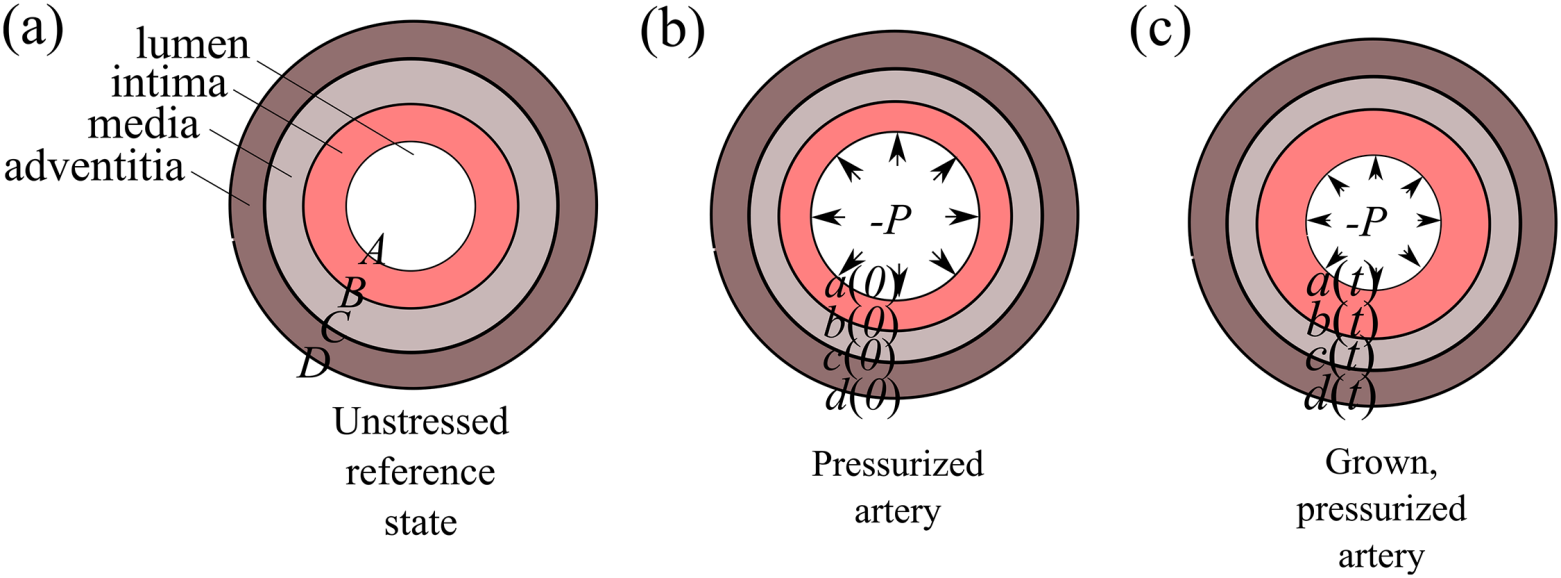
Multi-layer axisymmetric model for arterial remodeling. Geometry in the (a) reference, unstressed configuration when *t* < 0, (b) pressurized configuration at *t* = 0 and (c) grown, pressurized configuration for *t* > 0.

Our equations arise from applying the morphoelasticity framework [10, 11] to three concentric annuli: see Fig 2. While plaques are not usually concentric, this model is able to capture the important features of growth and remodeling. Specifically, we can use the model to understand the competition between growth (e.g. smooth muscle cells elaborating proteoglycans and collagen in the intima) and geometric constraints (e.g. the media mechanically restricting the growth of the intima). The delicate interplay between these two effects gives rise to unexpected results for arterial morphology and Glagov phenomena.

For *t* < 0, the artery starts in an unstressed reference state. At *t* = 0, we introduce a luminal pressure *P* > 0 and for *t* > 0, we allow the intima to undergo growth. The growth is assumed to be uniform throughout the intima and isotropic so that circumferential and radial growth are identical. The media and adventitia are modeled as strain-stiffening hyperelastic materials while the intima is described as a Neo-Hookean solid. Note that there is no growth or resorption in the media or adventitia. However, the media and adventitia can dilate in order to accommodate intimal expansion. In our model, a luminal encroachment (inward remodeling) is characterized by *a*(*t*) decreasing with *t* while luminal dilation (outward remodeling) is characterized by *a*(*t*) increasing with *t*.

The model that we employ here is a special case of the one in [12], except that we assume uniform growth in the intima and no homeostatic stresses. In the notation of [12], this corresponds to *ν*_1_ = *λ* = 0.

### Deformation and morphoelasticity decomposition

We consider a finite deformation where the cylinder can grow and deform while remaining cylindrical. We restrict ourselves to radial deformations uniform along the tube axis and only focus on the vessel’s cross section. We describe the deformation by the function

$$\begin{array}{c} r=r(R,t),\;A\leq R\leq D, \end{array}$$ (3)

where in the undeformed reference state, *A* is the lumen radius, *D* is the adventitia radius and *R* is the radial coordinate (see Fig 2). The deformation gradient tensor, **F** in cylindrical coordinates is given by

$$\begin{array}{c} F=\text{diag}(r^{\prime },r/R,1), \end{array}$$ (4)

and prime denotes differentiation with respect to *R*. The elastic tensor is given by **F**_e_ = diag(*α*_*r*_, *α*_*θ*_, *α*_*z*_) where *α*_*r*_, *α*_*θ*_ and *α*_*z*_ are geometric stretch factors in the radial, circumferential and axial directions respectively associated with the elastic deformation. Since we only consider radial deformations along the axis, we have *α*_*z*_ = 1. Because of the high water content of biological tissues, our vessel cross section can be assumed to be incompressible and we have det **F**_e_ = 1 which implies that *α*_*θ*_ ⋅ *α*_*r*_ = 1. Taking *α* = *α*_*θ*_, the elastic tensor is

$$\begin{array}{c} F_{e}=\text{diag}\left(\alpha^{-1},\alpha ,1\right). \end{array}$$ (5)

For the growth tensor, we assume that stretch factors in the radial and azimuthal directions associated with growth are the same so that

$$\begin{array}{c} F_{g}=\{\begin{array}{cc} \text{diag}(g,g,1), & A\leq R\leq B,\\ I, & B<R\leq D, \end{array} \end{array}$$ (6)

for some growth function *g*(*t*) (which we define later on) and **I** is the identity tensor.

The morphoelasticity assumption postulates that **F** = **F**_e_ **F**_*g*_, so in the intima

$$\begin{array}{c} r^{\prime }=\alpha^{-1}g\left(t\right), \end{array}$$ (7)

$$\begin{array}{c} r/R=\alpha g(t), \end{array}$$ (8)

which together imply that

$$\begin{array}{c} \frac{\partial r}{\partial R}=\frac{g^{2}\left(t\right)R}{r},\;A\leq R\leq B. \end{array}$$ (9)

In the media and adventitia,

$$\begin{array}{c} r^{\prime }=\alpha^{-1}, \end{array}$$ (10)

$$\begin{array}{c} r/R=\alpha , \end{array}$$ (11)

which together imply that

$$\begin{array}{c} \frac{\partial r}{\partial R}=\frac{R}{r},\;B\leq R\leq D. \end{array}$$ (12)

Therefore the deformation takes the form

$$\begin{array}{c} r\left(R,t\right)=\{\begin{array}{cc} {\left(a^{2}+g^{2}\left(t\right)\left(R^{2}-A^{2}\right)\right)}^{1/2}, & \;A\leq R\leq B,\\ {\left(b^{2}+R^{2}-B^{2}\right)}^{1/2}, & \;B\leq R\leq D. \end{array} \end{array}$$ (13)

from which we deduce

$$\begin{array}{c} b=r\left(B,t\right)={\left(a^{2}+g^{2}\left(t\right)\left(B^{2}-A^{2}\right)\right)}^{1/2}, \end{array}$$ (14)

$$\begin{array}{c} c=r\left(C,t\right)={\left(b^{2}+C^{2}-B^{2}\right)}^{1/2}, \end{array}$$ (15)

$$\begin{array}{c} d=r\left(D,t\right)={\left(b^{2}+D^{2}-B^{2}\right)}^{1/2}. \end{array}$$ (16)

Once *a* is given, *b*, *c* and *d* immediately follow from Eqs (14)–(16).

### Stress and equilibrium

We assume that growth is slow enough that the body is in mechanical equilibrium for all time. Balancing linear momentum and angular momentum, the Cauchy stress tensor **T** satisfies

$$\begin{array}{c} \nabla \cdot T=0. \end{array}$$ (17)

Assuming the material is incompressible, the Cauchy stress in the intima, media and adventitia is related to the elastic deformation **F**_e_ through

$$\begin{array}{c} T=F_{e}\frac{\partial W}{\partial F_{e}}-pI, \end{array}$$ (18)

where *p* is a Lagrange multiplier associated with the internal constraint of incompressibility, and *W* = *W*_1_, *W*_2_, *W*_3_ is the strain energy function for the intima, media and adventitia respectively. We assume that the intima is a Neo-Hookean material

$$\begin{array}{c} W_{1}\left(I_{1}\right)=\mu_{1}\left(I_{1}-3\right). \end{array}$$ (19)

For the media and adventitia, we use strain energy functions from [13]:

$$\begin{array}{ccc} W_{k}\left(I_{1},I_{4}^{\left(k\right)}\right) & = & \mu_{k}\left(I_{1}-3\right)+\frac{\eta_{k}}{\beta_{k}}\{e^{\beta_{k}[\left(1-\rho_{k}\right){\left(I_{1}-3\right)}^{2}+\rho_{k}{\left(I_{4}^{\left(k\right)}-1\right)}^{2}]}-1\}, \end{array}$$ (20)

$$\begin{array}{ccc} I_{1} & = & \alpha^{2}+\alpha^{-2}+1, \end{array}$$ (21)

$$\begin{array}{ccc} I_{4}^{\left(k\right)} & = & \alpha^{2}\cos^{2}\varphi_{k}+\sin^{2}\varphi_{k}, \end{array}$$ (22)

for some material parameters *μ*_*k*_, *η*_*k*_, *β*_*k*_, *ρ*_*k*_, *φ*_*k*_ and *k* = 2, 3. Mean values for these constants can be found by mechanical testing [13] and they are given in Table 1. The exponential term in Eq (20) accounts for the presence of collagen fibers which render the media and adventitia extremely resistant to large dilatations. If we let $\omega_{k}\left(\alpha \right)=W_{k}\left(I_{1}\left(\alpha \right),I_{3}^{\left(k\right)}\left(\alpha \right)\right)$, *k* = 1, 2, 3, then we can define auxiliary functions

$$\begin{array}{ccc} \omega_{1}\left(\alpha \right) & = & \mu_{1}\left(\alpha^{2}+\alpha^{-2}-2\right),\\ \omega_{k}\left(\alpha \right) & = & \mu_{k}\left(\alpha^{2}+\alpha^{-2}-2\right)+\frac{\eta_{k}}{\beta_{k}}\{e^{\beta_{k}[\left(1-\rho_{k}\right){\left(\alpha^{2}+\alpha^{-2}-2\right)}^{2}+\rho_{k}{\left(\alpha^{2}\cos^{2}\varphi_{k}+\sin^{2}\varphi_{k}-1\right)}^{2}]}-1\}, \end{array}$$

when *k* = 2, 3.

**Table 1:** Parameter values for a three-layer model of vessel remodeling.

Symbol	Meaning	Value	Reference
*P*	Pressure in coronary artery	50-110 mmHg	[14]
*μ*_1_	Intima material parameter	5 kPa	estimated
*μ*_2_	Media material parameter	1.27 kPa	[13]
*η*_2_	Media material parameter	21.60 kPa	[13]
*β*_2_	Media material parameter	8.21	[13]
*φ*_2_	Media fiber angle	20.61°	[13]
*ρ*_2_	Media material parameter	0.25	[13]
*μ*_3_	Adventitia material parameter	7.56 kPa	[13]
*η*_3_	Adventitia material parameter	38.57 kPa	[13]
*β*_3_	Adventitia material parameter	85.03	[13]
*φ*_3_	Adventitia fiber angle	67.0°	[13]
*ρ*_3_	Adventitia material parameter	0.55	[13]
Γ	Intima growth rate	0.5 year^−1^	-
*a*	Lumen radius in reference configuration	free parameter	-
*b* − *a*	Intima thickness in reference configuration	0.01 mm	estimated
*c* − *b*	Media thickness in reference configuration	0.32 mm	[13]
*d* − *c*	Adventitia thickness in reference configuration	0.34 mm	[13]

### Interface conditions

The only non-vanishing component of the mechanical equilibrium equation is

$$\begin{array}{c} \frac{\partial T_{rr}}{\partial r}+\frac{T_{rr}-T_{\theta \theta }}{r}=0, \end{array}$$ (23)

where *T*_*rr*_ and *T*_*θθ*_ are the radial and hoop Cauchy stresses. Then the stress-strain relationship Eq (18) implies that

$$\begin{array}{c} \frac{\partial T_{rr}}{\partial r}=\{\begin{array}{cc} \frac{\alpha }{r}\frac{\partial \omega_{k}}{\partial \alpha }, & a<r<b,\\ \frac{\alpha }{r}\frac{\partial \omega_{k}}{\partial \alpha }, & b<r<c,\\ \frac{\alpha }{r}\frac{\partial \omega_{k}}{\partial \alpha }, & c<r<d. \end{array} \end{array}$$ (24)

We assume zero traction on the outer boundary *T*_*rr*_(*r* = *d*) = 0 and a luminal pressure *P* at the inner boundary so *T*_*rr*_(*r* = *a*) = −*P*. Using these conditions and integrating Eq (24), we obtain the radial stress as

$$\begin{array}{c} T_{rr}\left(r\right)=\{\begin{array}{cc} -P+\int_{a}^{r}\frac{dr}{r}2\mu_{1}\left(\alpha^{2}-\alpha^{-2}\right), & a\leq r\leq b,\\ -\int_{c}^{d}\frac{dr}{r}\{2\mu_{3}\left(\alpha^{2}-\alpha^{-2}\right)+4\eta_{3}\alpha^{2}F\left(\alpha ;\varphi_{3},\rho_{3}\right)e^{\beta_{3}G\left(\alpha ;\varphi_{3},\rho_{3}\right)}\} & \\ \;-\int_{r}^{c}\frac{dr}{r}\{2\mu_{2}\left(\alpha^{2}-\alpha^{-2}\right)+4\eta_{2}\alpha^{2}F\left(\alpha ;\varphi_{2},\rho_{2}\right)e^{\beta_{2}G\left(\alpha ;\varphi_{2},\rho_{2}\right)}\}, & b\leq r\leq c,\\ -\int_{r}^{d}\frac{dr}{r}\{2\mu_{3}\left(\alpha^{2}-\alpha^{-2}\right)+4\eta_{3}\alpha^{2}F\left(\alpha ;\varphi_{3},\rho_{3}\right)e^{\beta_{3}G\left(\alpha ;\varphi_{3},\rho_{3}\right)}\}, & c\leq r\leq d. \end{array} \end{array}$$ (25)

where

$$\begin{array}{ccc} F(\alpha ;\varphi_{k},\rho_{k}) & = & \left(1-\rho_{k}\right)\left(\alpha^{2}+\alpha^{-2}-2\right)\left(1-\alpha^{-4}\right)+\rho_{k}\left(\alpha^{2}\cos^{2}\varphi_{k}+\sin^{2}\varphi_{k}-1\right)\cos^{2}\varphi_{k},\\ G(\alpha ,\varphi_{k},\rho_{k}) & = & \left(1-\rho_{k}\right){\left(\alpha^{2}+\alpha^{-2}-2\right)}^{2}+\rho_{k}{\left(\alpha^{2}\cos^{2}\varphi_{k}+\sin^{2}\varphi_{k}-1\right)}^{2}. \end{array}$$

Continuity of radial stress at *r* = *c* is automatically satisfied when *T*_*rr*_ takes the form above. Continuity of radial stress at *r* = *b* implies

$$\begin{array}{ccc} -P+\int_{a}^{b}\frac{dr}{r}2\mu_{1}\left(\alpha^{2}-\alpha^{-2}\right) & = & -\int_{c}^{d}\frac{dr}{r}\{2\mu_{3}\left(\alpha^{2}-\alpha^{-2}\right)+4\eta_{3}\alpha^{2}F\left(\alpha ;\varphi_{3},\rho_{3}\right)e^{\beta_{3}G\left(\alpha ;\varphi_{3},\rho_{3}\right)}\}\\ & & \;-\int_{b}^{c}\frac{dr}{r}\{2\mu_{2}\left(\alpha^{2}-\alpha^{-2}\right)+4\eta_{2}\alpha^{2}F\left(\alpha ;\varphi_{2},\rho_{2}\right)e^{\beta_{2}G\left(\alpha ;\varphi_{2},\rho_{2}\right)}\}, \end{array}$$ (26)

where the elastic stretch is

$$\begin{array}{c} \alpha \left(R,t\right)=\{\begin{array}{cc} \frac{r}{gR}=\frac{\sqrt{a^{2}+g^{2}\left(t\right)\left(R^{2}-A^{2}\right)}}{g(t)R}, & A\leq R\leq B,\\ \frac{r}{R}=\frac{\sqrt{b^{2}+R^{2}-B^{2}}}{R}, & B\leq R\leq D. \end{array} \end{array}$$ (27)

Furthermore, since $\frac{dr}{r}=\frac{1}{\alpha^{2}}\frac{dR}{R}$ using Eqs (7), (8), (10), (11) and (26) can be recast in terms of reference frame variables:

$$\begin{array}{ccc} & & -P+\int_{A}^{B}\frac{dR}{R}2\mu_{1}\left(1-\alpha^{-4}\right)=\\ & & \;-\int_{B}^{C}\frac{dR}{R}\{2\mu_{2}\left(1-\alpha^{-4}\right)+4\eta_{2}F\left(\alpha ;\varphi_{2},\rho_{2}\right)e^{\beta_{2}G\left(\alpha ;\varphi_{2},\rho_{2}\right)}\}\\ & & \;-\int_{C}^{D}\frac{dR}{R}\{2\mu_{3}\left(1-\alpha^{-4}\right)+4\eta_{3}F\left(\alpha ;\varphi_{3},\rho_{3}\right)e^{\beta_{3}G\left(\alpha ;\varphi_{3},\rho_{3}\right)}\}. \end{array}$$ (28)

Providing that *g*(*t*) is given (see below), Eq (28)—with *α* defined in Eq (27) – along with Eqs (14)–(16) constitute four equations for four unknowns *a*(*t*), *b*(*t*), *c*(*t*) and *d*(*t*).

### Growth function

Our model is completed upon specifying the growth function *g*(*t*). Suppose the intima grows at a constant rate Γ, which is a result of cell proliferation and production of extracellular matrix by smooth muscle cells. Then conservation of mass implies [10]

$$\begin{array}{c} \text{Trace}(F_{g}^{-1}{\dot{F}}_{g})=\Gamma . \end{array}$$ (29)

Furthermore, assume that this growth rate is uniform throughout the intima so Γ is a constant with respect to both space and time. Since we have assumed that the geometric stretch associated with growth is the same in the radial and azimuthal directions, the growth rate Γ uniquely determines the growth function *g*:

$$\begin{array}{c} g\left(t\right)=\exp\{\frac{\Gamma t}{2}\}. \end{array}$$ (30)

When presenting our results in the next section, we often use stenosis, as opposed to time, to measure disease progression. For these plots, the value of Γ is unimportant in the following sense: if Γ is small, it takes a longer time to reach a particular stenosis value. Likewise, if Γ is large, it just takes a shorter time to reach the same value. The model produces the same vessel configuration for a given stenosis, irrespective of the value of Γ. Occasionally, we show evolutions of wall radii with respect to time, measured in years. In this case, we take Γ = 0.5 year^−1^: see Table 1.

In practice, the rate at which plaque grows in arteries is highly variable, depending on lifestyle choices, environment and genetics (a reasonable way to model this situation is to have Γ drawn from a probability distribution). However, in light of the discussion above, plots of the lumen area against stenosis are invariant with respect to Γ. Under the assumption Eq (30), Glagov’s representation of disease progression (Fig 1) is not affected by how quickly the plaque grows and the precise value of Γ is inconsequential.

## Results

### Glagov Remodeling

Glagov’s data was obtained from cross sections of coronary arteries harvested post-mortem. To simulate this situation, we first ran our model with the parameters in Table 1 and a luminal pressure of *P* = 50 − 110 mmHg. After the simulation was completed, for each time point, we unloaded the vessel by taking *P* = 0 to find dimensions corresponding to unpressurized vessels. Because growth in our model is stress-independent, these vessel dimensions are identical to just simulating growth with *P* = 0 for *t* ≥ 0.

**Fig 3:**
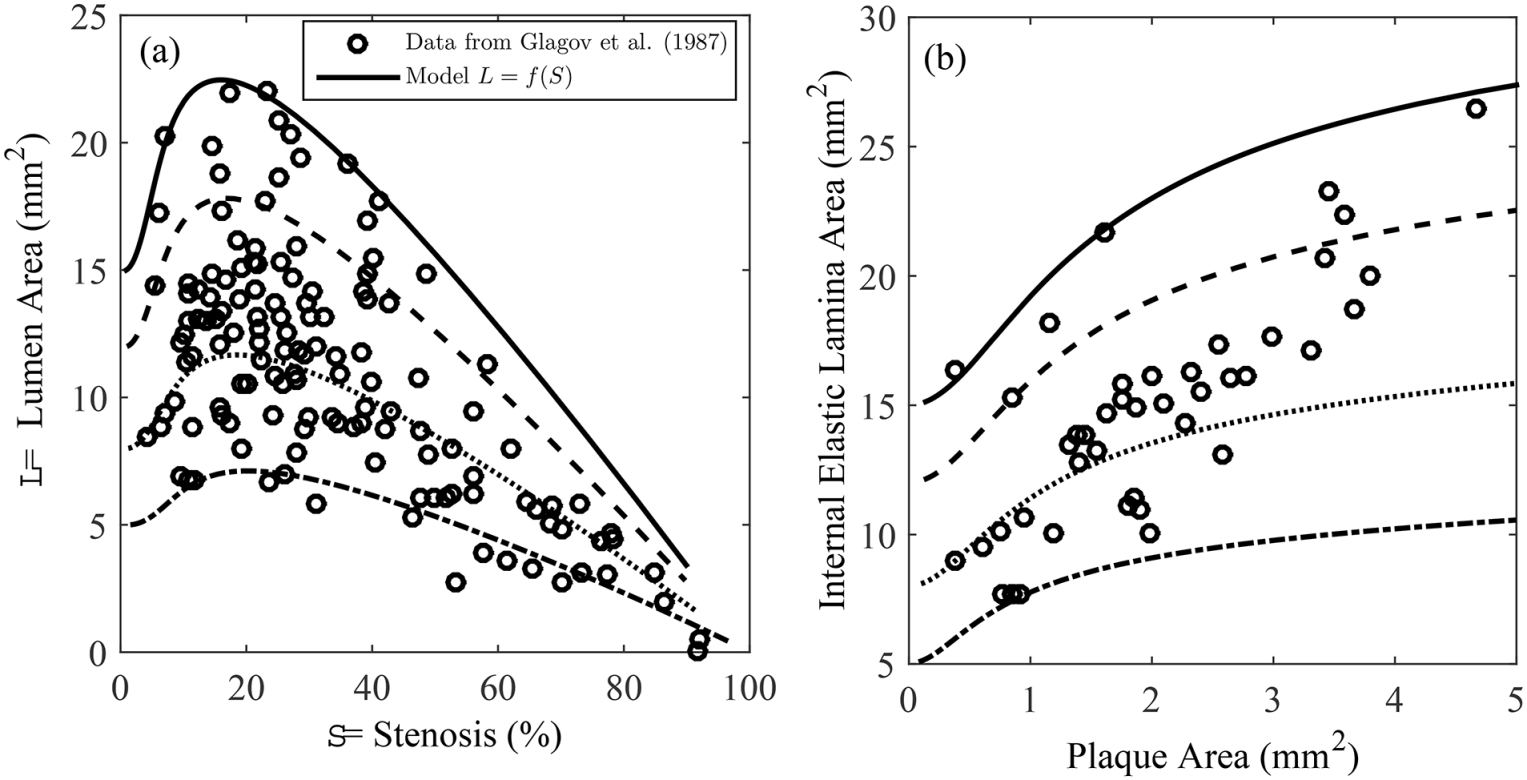
Comparison of model results to Glagov’s data. (a) Data from Glagov’s paper of lumen area *L* versus stenosis fraction *S*. Superimposed are solid curves *L* = *f*(*S*), generated by our model initialized with reference lumen areas *πa*(0)^2^ = *πA*^2^ = 5 (dash-dotted), 8 (dotted), 12 (dashed) and 15 (solid) mm^2^. (b) Data and model prediction of the internal elastic lamina area *πb*^2^ as a function of plaque area *π*(*b*^2^ − *a*^2^).

Our model is able to reproduce Glagov phenomena in the following sense: curves of the lumen area *L* against stenosis fraction *S*, described by *L* = *f*(*S*) are non-monotonic, exhibiting a maximum at *S* = S*: see Fig 3(a). In this paper, we call S* the critical stenosis when S* ∈ (0, 1) and

$$\begin{array}{c} f^{\prime }\left(S^{*}\right)=0, \end{array}$$ (31)

$$\begin{array}{c} f^{\prime \prime }\left(S^{*}\right)<0. \end{array}$$ (32)

In Fig 3, we initialize our simulations with 4 different values for the undeformed lumen area *πA*^2^. In (a) we superimpose our predictions with Glagov’s data, which was extracted from his paper using the program WebPlotDigitizer (arohatgi.info/WebPlotDigitizer/). The general trend for this range of initial conditions is for the lumen area to increase before decreasing. This is consistent with Glagov’s hypothesis that vessels undergo outward remodeling before encroachment of the lumen. In (b) we show the increase of the internal elastic lamina area as the plaque area increases. Again, these curves are superimposed on the data taken from Glagov’s paper. Our remodeling simulations of an unloaded vessel suggest that the critical stenosis, as defined by Eqs (31) and (32), is about 20%, which is smaller than the generally accepted value of 40%, first suggested by Glagov.

### Remodeling in-vivo and ex-vivo

The PROSPECT (Providing Regional Observations to Study Predictors of Events in the Coronary Tree) trial [15] was a large multi-center trial that attempted to elucidate the natural history of coronary atherosclerosis. One of the data sets consisted of measurements of lumen area and stenosis fractions for the left main coronary artery of 552 patients [8]. These measurements were used to see if Glagov remodeling occurred *in-vivo*. The data was first split into two sets: those with <40% stenosis and those with >40% stenosis. Upon performing linear regression on both sets, the authors found that the lumen area decreased at a slower rate (with respect to stenosis) when stenosis fraction was <40%, while the rate of decrease was faster when stenosis fraction was >40%. There appeared to be little evidence to support a compensating or expanding lumen. Thus the remodeling seems to be qualitatively different than the sequence of events portrayed in Fig 1.

**Fig 4:**
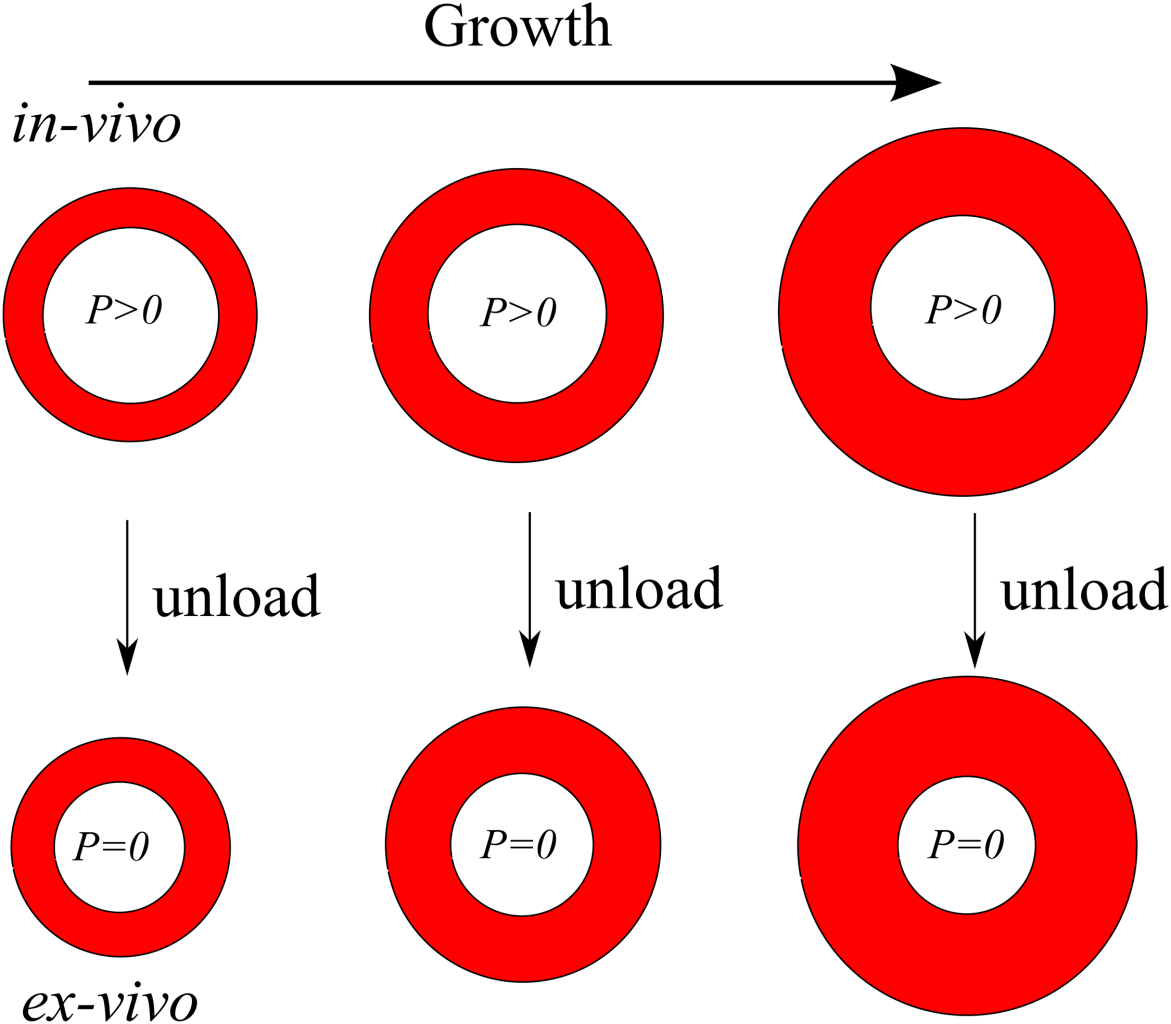
Growth of the intima *in-vivo* and *ex-vivo*. *Ex-vivo* configurations are quickly obtained in our model either by taking *P* = 0 at each step of the *in-vivo* growth or taking *P* = 0 from the start of the simulation.

The contrasting data sets from the PROSPECT trial and Glagov’s paper motivate us to consider the following question: does the remodeling, quantified by the *L* = *f*(*S*) curve in Fig 3(a) differ *in-vivo* and *ex-vivo*? In both cases, the intima is undergoing growth but when vessel sections are examined post-mortem, the lumens are evacuated and depressurized, resulting in a contraction of the arterial cross-section: see Fig 4. Intuitively, we would expect that vessel dimensions should be different depending on whether the vessels are studied *in-vivo* or *ex-vivo*: see Table 2. Note that in our model, plaque area *ex-vivo* and *in-vivo* are the same because of the incompressibility assumption. As a result, we expect vessel stenoses to be lowered when measured *in-vivo*. In our model, we are able to convert from *in-vivo* vessel dimensions to *ex-vivo* by changing *P* = 50 − 110 mmHg to *P* = 0 mmHg.

**Table 2:** Qualitative differences in vessel geometry for *ex-vivo* and *in-vivo* vessels. Stenosis fraction is defined in Eq (2).

	*ex-vivo*	*in-vivo*
IEL area	Smaller	Larger
Lumen area	Smaller	Larger
Plaque area	Same	Same
Stenosis	Larger	Smaller

**Fig 5:**
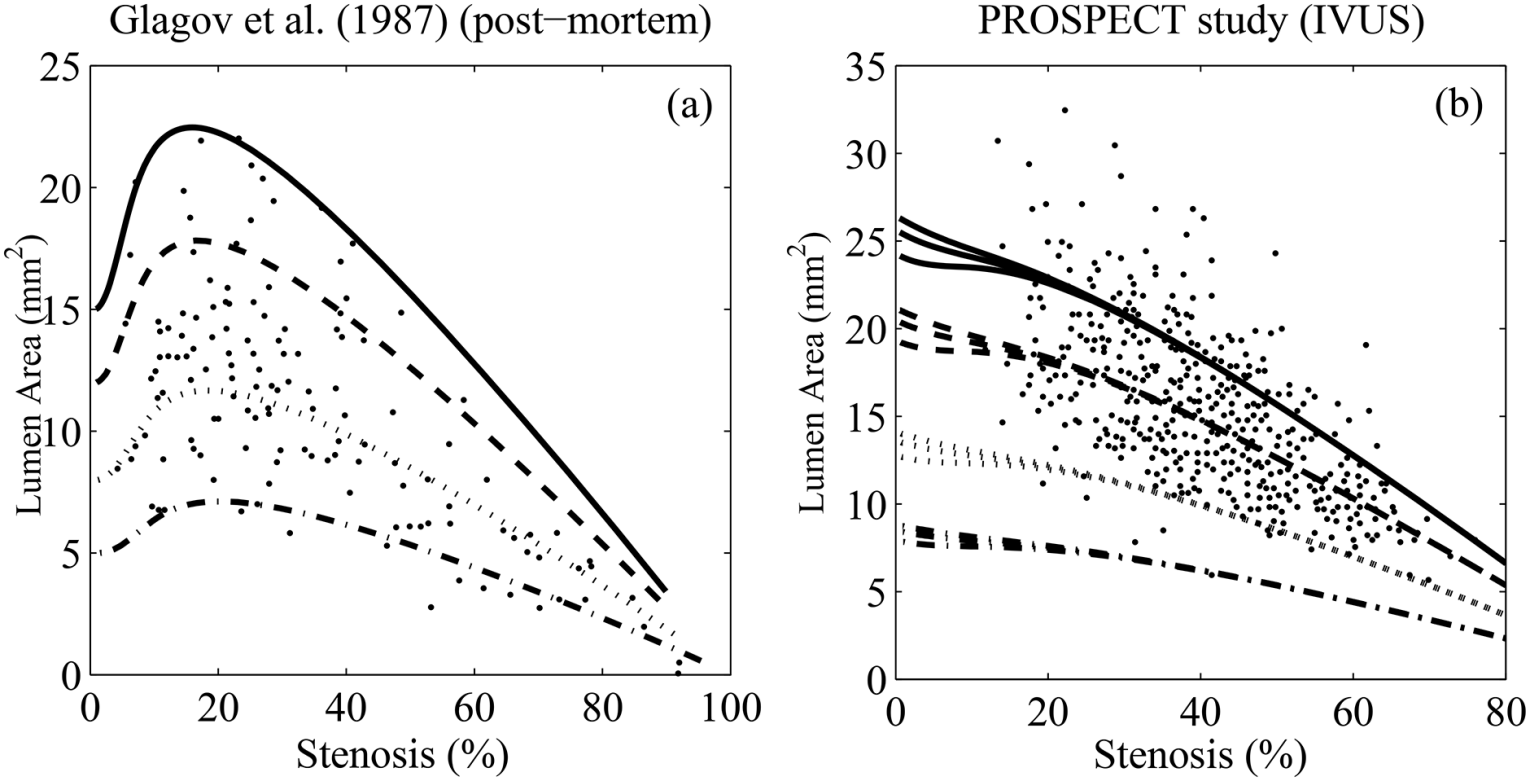
Comparison of *ex-vivo* and *in-vivo* vessel dimensions from (a) Glagov’s paper [4] and (b) the PROSPECT trial [8]. Four model vessel cross-sections are simulated with unloaded initial lumen areas of 5, 8, 12 and 15 mm^2^ in (a). The growth of corresponding vessels loaded with pressure *P* = 50, 80, 110 mmHg is simulated in (b) (for a fixed stenosis, lumen area increases as *P* increases). The *ex-vivo* vessels in (a) are predicted to exhibit a local maximum with respect to stenosis fraction, while the equivalent, loaded lumen areas of *in-vivo* vessels in (b) are monotonically decreasing with respect to stenosis.

Our mathematical model predicts a qualitative difference in the *L* = *f*(*S*) curve depending on whether *P* = 0 mmHg or *P* = 50 − 110 mmHg: see Fig 5. For the parameter values in Table 1, we found that pressurized vessels did not exhibit critical stenoses and this behavior is insensitive to the luminal pressure. However, the qualitative aspect of Glagov’s phenomenon is robust in the sense that the lumen area decreases slowly when the stenosis fraction is small, and decreases more quickly when the stenosis fraction is large. The lumen pressure *P* should be thought of as an average of diastolic and systolic coronary pressures [14].

**Fig 6:**
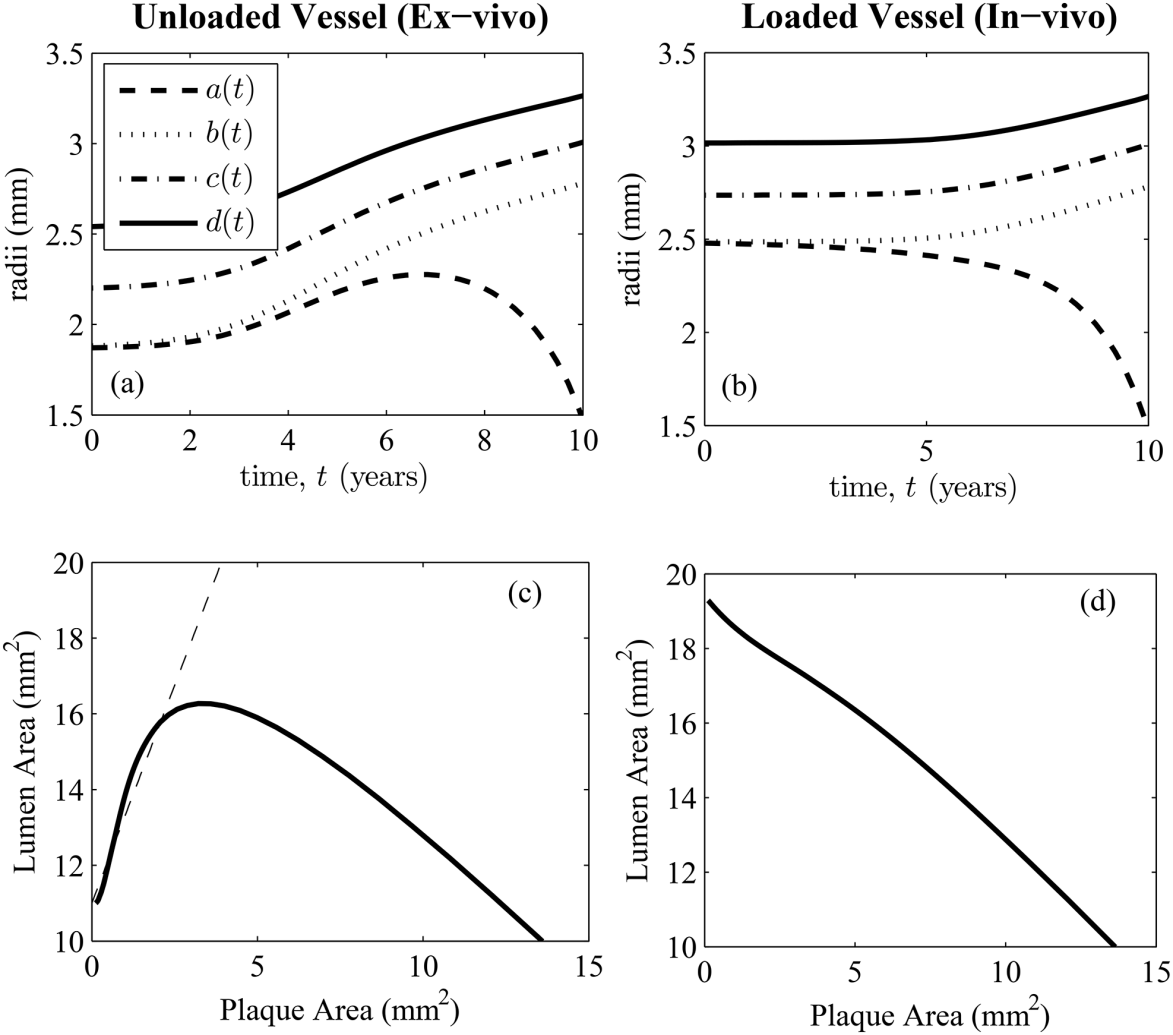
Remodeling of *ex-vivo* and *in-vivo* vessels. (a) and (b): Time evolution of *ex-vivo* and *in-vivo* vessels as they undergo identical uniform growth in the intima. The *ex-vivo* vessel is unpressurized with *P* = 0 while the *in-vivo* vessel is subject to a luminal blood pressure of *P* = 110 mmHg. (c) and (d) are the respective plots of lumen area vs. plaque area which are qualitatively different. Glagov’s *ex-vivo* data suggested that during the early stages of atherosclerosis, the lumen area increased by 2.3 mm^2^ for every mm^2^ growth of plaque (dashed line in (c)).

Another qualitative difference between *ex-vivo* and *in-vivo* vessels is manifested when we study how the lumen area changes with plaque area: see Fig 6. For *ex-vivo* vessels, the trend is non-monotonic, with the lumen area exhibiting a maximum, while for *in-vivo* vessels, lumen area decreases monotonically with respect to plaque area. Note that in these plots, the horizontal axis represents plaque area, not stenosis. This method of quantifying vessel remodeling was also performed in [4]. Specifically, Glagov took his original data set and disregarded samples that had stenoses >20%. He analyzed the remaining mildly stenotic plaques and concluded that during the early stages of the disease, the lumen area tended to “over-compensate” in the sense that it expanded about 2.3 mm^2^ for every 1 mm^2^ increase in the plaque area. Interestingly, our mathematical model is able to reproduce this result when the plaque area is small: see Fig 6(c). The dashed line with slope 2.3 represents Glagov’s observation. This result highlights the danger of trying to predict remodeling behaviors by studying curves of lumen area against plaque area and/or stenoses: the behavior can be very different depending on whether the samples are studied *ex-vivo* or *in-vivo*.

**Fig 7:**
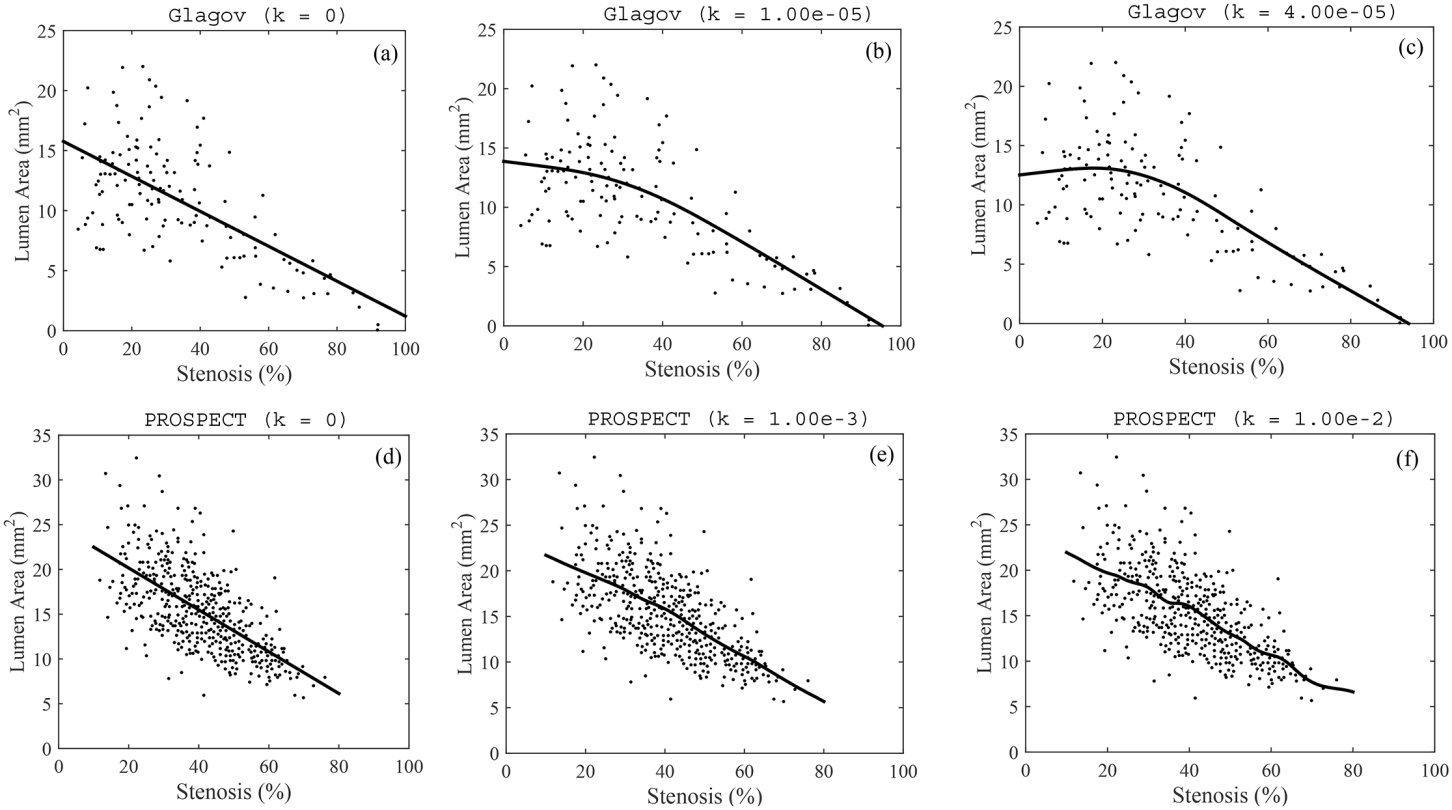
Best-fit smoothing cubic-splines through Glagov’s *ex-vivo* data (top row) and *in-vivo* data from the PROSPECT trial (bottom row). In each case, more curvature is introduced into the best-fit curve by allowing *k* to increase: see Eq (33). A critical stenosis emerges for Glagov’s data, but not for the PROSPECT trial.

So far, we have deduced features of the vessel’s evolution from the mathematical model independently of the data itself. We now turn our attention to the data from Glagov’s paper and the PROSPECT trial. Do the data sets support our earlier conclusions? Namely, that (i) the critical stenosis for *ex-vivo* data is around 20% rather than 40%, (ii) *ex-vivo* lumen areas increase then decrease with respect to stenosis and (iii) *in-vivo* lumen areas are monotonically decreasing with respect to stenosis. In Fig 7 we use smoothing splines from the Matlab computational environment to fit the data from Glagov’s paper and from the PROSPECT trial. For data sets (*s*_*i*_, *ℓ*_*i*_), *i* = 1, …, *N*, Matlab’s smoothing splines are cubic splines *L*(*S*) that minimize the functional

$$\begin{array}{c} k\sum_{i=1}^{N}{\left(\ell_{i}-L\left(s_{i}\right)\right)}^{2}+\left(1-k\right)\int_{\ell_{1}}^{\ell_{N}}{\left(\frac{d^{2}L}{dS^{2}}\right)}^{2}dS, \end{array}$$ (33)

where 0 ≤ *k* ≤ 1. The first term penalizes deviations from the data while the second term penalizes deviations from a straight line. When *k* → 1, the resulting spline passes through all data points, whereas when *k* → 0, the resulting spline is a best-fit straight line in the least-squares sense [16].

We increased *k* (which gradually introduces more curvature into the best-fit curve) on both data sets and found that for Glagov’s data, an interior maximum did emerge for sufficiently large *k* values, and the location of the interior maximum was at about 20%, consistent with the results from our modeling efforts: see Fig 7. On the other hand, although the best-fit curve for the PROSPECT data did develop small “bumps,” as *k* increased, there was never any evidence of a critical stenosis and we could not detect any systematic, large scale features in the data set.

## Discussion

Understanding Glagov remodeling from mechanical and biological standpoints is important for understanding the progression of atherosclerosis. Glagov originally hypothesized that critical stenosis in coronary vessels occured at 40% but his prediction was for depressurized vessels harvested post-mortem. Although this paradigm has been extended to *in-vivo* vessels in a large human study [15], a more critical analysis of Glagov’s original data and the results of our mathematical model show that first, if a critical stenosis does occur *ex-vivo*, it occurs at 20%, not 40%. Second, remodeling *in-vivo* and *ex-vivo* are qualitatively different: for *in-vivo* vessels, there is no critical stenosis; rather the lumen area decreases slowly for small stenoses and more quickly for large stenoses (see Fig 5). This behavior appears to be robust with respect to changes in the lumen pressure and initial lumen area.

How can we understand *ex-vivo* Glagov remodeling from a physical standpoint? In our model, we think that the lumen area initially increases because the media and adventitia are compliant relative to the intima. The result is for the lumen and IEL radii to increase. Once the media and adventitia are sufficiently strained, the collagen fibers within these layers become extremely stiff with respect to extension. These outer layers cannot undergo further dilatation, acting like a straight jacket for the intima [17], so that during the later stages of growth, only inward remodeling can occur. This qualitative explanation for Glagov remodeling could explain the non-monotonicity of the curves in Fig 3(a). However, changes in vessel dimensions due to shear-stress [18, 19] and atrophy in the media [4] could also play important roles.

The strain-energy density functions and associated parameters are important parts of our model. In our paper, we employ a Neo-Hookean energy function for the intima following [20], and an anisotropic Fung-type energy function for the media and adventitia. The parameter values for the media and adventitia were taken from healthy arteries. For the intima, we used a value of *μ*_1_ = 5 kPa, which is on the low side, even for healthy arteries. However, this value results in a remodeling behavior that has two desirable properties. First, it gives a critical stenosis that agrees with the one predicted by spline fitting in Fig 7. Second, it results in the lumen area initially increasing by roughly 2.3 mm^2^ for every 1 mm^2^ increase in plaque area: see Fig 6(c). We did try increasing *μ*_1_ to 27 kPa, following [13] but the resulting vessel evolution loses both of these properties.

Are these strain energy functions reasonable? There are two main issues. The first is using a Neo-Hookean material to approximate the mechanical properties of the intima. A Neo-Hookean material is isotropic, and it appears that healthy intimas are generally anisotropic [13]. However, mechanical tests on fibrotic intimas in [17] suggest that the intima becomes more isotropic as the disease progresses and lends some credence to our approach. This could be because as the intima becomes more fibrotic, the ratio of smooth muscle cells to collagen fibers increases and anistropy plays a smaller role. In future work, this effect could be mimicked by using an exponential strain energy function like Eq (20) for the intima, and taking *ρ*_1_ → 0 as *t* increases.

The second issue is using the same energy functions (and parameters) for the vessel layers even as the disease progresses. For the media and adventitia, this assumption is probably reasonable since their histology changes relatively little compared to the intima [21]. However, there is general agreement from the bioengineering community [17, 22, 23] that the intima becomes stiffer as it becomes more fibrotic. While this effect is not included in the sense that *μ*_1_ is time-independent in our model, we note that the stiffness of our model intima does change because it is growing. Actually, quantifying the stiffness of the model intima when *t* > 0 can be challenging since stiffness moduli are usually defined for linearly elastic materials. One way of proceeding is to take a grown configuration, study how stresses behave with respect to small deformations, and deduce the incremental modulus of the intima at different times [24]. Again we leave this for future work.

## Conclusions

In this paper, we proposed a mathematical model for vessel growth and remodeling that is based on 3 hyperelastic annuli representing the intima, media and adventitia. Our paper contains two main results. First, both the model and a direct analysis of Glagov’s data by smoothing splines suggest that the critical stenosis is around 15-20% rather than 40%. Hence both our model and the data independently suggest that the critical stenosis—if it exists—is significantly lower than the value given in Glagov’s original paper. Our second result concerns the difference in Glagov remodeling *ex-vivo* versus *in-vivo*. A standard way to quantify the progression of disease in arteries is to plot the lumen area as a function of stenosis fraction. In this paper we showed that there are qualitative differences in this curve depending on whether the measurements are taken *ex-vivo* or *in-vivo*. Specifically, while the lumen area vs. stenosis fraction curve *ex-vivo* contains an interior maximum and presents a critical stenosis, the same curve *in-vivo* is monotonically decreasing. We advocate that care should be taken when making conclusions about how blood vessels remodel *in-vivo* by studying *ex-vivo* cross-sections.

We caution that our conclusions are still tentative at this stage since there are several limitations to our model. First, our model is geometrically axisymmetric while many plaques exhibit an eccentric cross-sectional morphology. Second, we do not account for biologically important phenomena such as vasodilator release and auto-regulatory mechanisms for vessel size adjustment: the results presented here are a consequence of mechanics and growth only. This could be one reason why we had to assume an overly-compliant intima in order for the model to give a critical stenosis of 20% (consistent with spline-fitting in Fig 7) and the correct over-compensation behavior for early-stage plaques. Finally, we assumed that the intima was a strain-softening Neo-Hookean material even though plaques generally get stiffer as they become more fibrotic.

Nevertheless, we think our model is a stepping stone towards a more fundamental understanding of Glagov remodeling. While our quantitative predictions are for coronary arteries, the model could predict the remodeling behavior in other types of arteries, provided it is initialized with the appropriate geometric and material parameters. To our knowledge, a rigorous explanation of Glagov remodeling has never been attempted, although in his paper, Glagov thought that failure of a compensating lumen (i.e. the switch from compensating to inward remodeling) was due to a decreased sensitivity of the endothelium to wall shear stress [18, 19]. Media atrophy could also play an important role as the lumen enlarges. It would be interesting to see if incorporating these richer biological features into our model changes the critical stenosis or the qualitative differences between *in-vivo* and *ex-vivo* remodeling.

Another reason for interpreting our conclusions cautiously is that the patients in the PROSPECT trial were older men aged 50.2-67.3 years. As a result, it is not surprising that there were basically no plaques in this study that had a stenosis fraction less than 10%. In contrast, Glagov’s data set was comprised of patients who were between the ages of 18 and 98 years and there were several plaques that were in their early stages. Because of this discrepency, the conclusions we draw from fitting smoothing cubic splines in Fig 7 are tentative and ideally we would want to repeat the analysis on a fuller *in-vivo* data set with more early-stage plaques.

In the clinical context, our results suggest that it is important to diagnose intimal encroachment in the first phase of remodeling before a rapid decline in flow capacity occurs (recall that although there is no critical stenosis in *in-vivo* vessels, there is a slow decline in lumen area followed by a more rapid one: see Fig 5). Results like Fig 6 provide some guidance for how much time the vessel spends in each of the phases and the time clinicians have to diagnose a plaque in its early stages. Our results could also be used to help develop risk measures in terms of specific geometric measurements of the vessel cross-section. For example, a mathematical model along with some longitudinal measurements of lumen area could be used to predict future remodeling behavior and/or the time taken for stenosis to fall below a certain level.
